# Eluxadoline Versus Antispasmodics in the Treatment of Irritable Bowel Syndrome: An Adjusted Indirect Treatment Comparison Meta-analysis

**DOI:** 10.3389/fphar.2022.757969

**Published:** 2022-02-23

**Authors:** Di Qin, Qing-Feng Tao, Shi-Le Huang, Min Chen, Hui Zheng

**Affiliations:** ^1^ Third Hospital/Acupuncture and Tuina School, Chengdu University of Traditional Chinese Medicine, Chengdu, China; ^2^ Acupuncture department, Hospital of Chengdu University of Traditional Chinese Medicine, Chengdu, China; ^3^ Department of Colorectal Diseases, Hospital of Chengdu University of Traditional Chinese Medicine, Chengdu, China

**Keywords:** eluxadoline, antispasmodics, irritable bowel syndrome, meta-analysis, abdominal pain

## Abstract

**Objective:** Eluxadoline is a newly approved drug for irritable bowel syndrome (IBS), but it has rarely been compared with positive controls. We aimed to compare eluxadoline with antispasmodics in the treatment of IBS.

**Methods:** We searched the OVID Medline, Embase, and the Cochrane Central Register of Controlled Trials databases for randomized controlled trials (RCTs) comparing eluxadoline or antispasmodics with placebo. The search was conducted from 1 January 1980, to 1 September 2020, without any language restrictions. The primary efficacy outcome was the relief of abdominal pain, defined by a reduction of pain scores of at least 30% from baseline. The secondary efficacy outcome was the relief of global IBS symptoms, defined by a composite response of a decrease in abdominal pain and improvement in stool consistency on the same day for at least 50% of the days assessed. The data were pooled using a random-effects model. Outcome estimates were pooled by using Risk Ratios (RRs) and P-scores.

**Results:** Forty-two trials with 8,457 participants were included from 45 articles. Compared with placebo, each of drotaverine, pinaverium, alverine combined with simethicone (ACS) and eluxadoline 100 mg was highly effective in the relief of abdominal pain, with drotaverine [RR, 2.71 (95% CI, 1.70 to 4.32), P-score = 0.95] ranking first. Drotaverine, otilonium, cimetropium, pinaverium, and eluxadoline 100 mg had significantly high the relief of global IBS symptomss, for which drotaverine [RR, 2.45 (95% CI, 1.42 to 4.22), P-score = 0.95] was ranked first. No significant difference was found between these interventions. Pinaverium had a significantly higher the relief of global IBS symptoms than eluxadoline [RR, 1.72 (95% CI, 1.33 to 2.21)] on sensitivity analysis. However, no significant difference was found in the number of adverse events between each intervention and the placebo.

**Conclusion:** Our network meta-analysis showed that eluxadoline 100 mg was at least as effective as antispasmodics in relieving abdominal pain in IBS. But eluxadoline had more reported adverse events. Antispasmodics are still the first choice for the treatment of IBS.

## 1 Introduction

Irritable bowel syndrome (IBS) is a common functional bowel disorder mainly characterized by recurrent abdominal pain associated with defecation and changes in stool form or frequency (Black et al., 2020b). And affects 10% of people worldwide (Black et al., 2020a). IBS is classified into four subtypes according to abnormal bowel habits: diarrhea-predominant IBS (IBS-D), constipation-predominant IBS (IBS-C), mixed-type IBS (IBS-M), or unclassified IBS (Lacy et al., 2016). In a meta-analysis of 14 studies of patients with IBS, IBS-D was the most prevalent, accounting for 40.0% of the patient population (Lovell and Ford, 2012). The recurrent gastrointestinal symptoms of IBS affect patients’ sleep and personal relationships (Hulisz, 2004), contributing to anxiety and depression (Kovács and Kovács, 2007; Buono et al., 2017), all of which lower the quality of life, work efficiency, and social production efficiency and increase the social medical burden (Spiegel, 2009; Ford et al., 2017). Moreover, researches show the severity of IBS correlated positively with occupational stress, and both were negatively associated with workability (Buselli et al., 2021). Although novel therapies for IBS continue to be developed, many doctors prefer traditional therapies, such as soluble fiber, antispasmodic drugs, peppermint oil, and gut-brain neuromodulators (Enck et al., 2016; Camilleri, 2018; Black et al., 2020b).

Currently, symptomatic treatment is mainly used, and pharmacological treatments are the primary choice for relieving IBS symptoms. Antispasmodics are considered the first-line medical treatment for IBS (Moayyedi et al., 2019) and include drugs that have anticholinergic or calcium channel-blocking properties (Chey et al., 2015). Antispasmodics relieve pain and improve bowel habits by inhibiting contractile pathways in the gut wall and changing transit time in the colon (Martínez-Vázquez et al., 2012). Antispasmodics are available for all subtypes of IBS (Lacy et al., 2016) and are better than placebo in preventing recurrence of IBS symptoms (Ford et al., 2018b). However, patients taking antispasmodics were more likely to experience adverse events than the placebo group, with dry mouth, fatigue, drowsiness, constipation, dizziness, and blurred vision being the most common (Chey et al., 2015; Ford et al., 2018b). The low levels of satisfaction with conventional medicines have caused an increasing number of patients and physicians to seek alternative therapies.

Eluxadoline is an orally administered, minimally absorbed agent that acts locally in the gastrointestinal tract as a mixed µ-opioid receptor agonist and δ-opioid receptor antagonist (Davenport et al., 2015; Keating, 2017; Özdener and Rivkin, 2017). Eluxadoline can relieve abdominal pain and diarrhea by slowing gastrointestinal motility and decreasing visceral hypersensitivity (Lembo et al., 2016) and is commonly used in the treatment of IBS-D (Keating, 2017). Several systematic reviews and randomized controlled trials (RCTs) found that eluxadoline 100 mg resulted in greater improvement of IBS symptoms, abdominal pain, and quality of life (Dove et al., 2013a; Lembo et al., 2016; Black et al., 2020a). The most commonly reported adverse reactions are constipation, nausea, and pancreatitis (Dove et al., 2013a; Cash et al., 2017).

At present, antispasmodics are one of the conventional first-line drugs commonly prescribed IBS. Antispasmodics have obvious advantages in relieving pain. However, a comparison of the efficacy of eluxadoline with antispasmodics in relieving abdominal pain has not been conducted, and head-to-head comparison trials are therefore warranted. Indirect treatment comparison (ITC) meta-analysis can compare the relative effects of two treatments under the condition that they have a common comparator–placebo or active control, in the absence of head-to-head comparison studies. Therefore, we conducted a network meta-analysis to compare the effectiveness and adverse events of eluxadoline and antispasmodics in treating IBS symptoms. The primary aim was to establish whether eluxadoline was of comparable efficacy to antispasmodics in relieving abdominal pain in IBS.

## 2 Materials and Methods

### 2.1 Study Source

The study was following the Preferred Reporting Items for Systematic Reviews and Meta-Analyses statement and its extension for network meta-analysis (Page et al., 2021). OVID Medline, Embase, and the Cochrane Central Register of Controlled were searched from inception to 1 September 2020. Screening randomized controlled trials compared eluxadoline or antispasmodics with placebo or one of antispasmodics in the management of IBS. Clinical registries (Clinicaltrials.gov) were searched for that were completed but unpublished. Studies on IBS were identified with the terms: irritable bowel syndrome and IBS. Other terms included randomized controlled trial, controlled trial, mebeverine, trimebutine, drotaverine, eluxadoline, etc., Search strategies were showed in Supplementary Table S2.

### 2.2 Study Selection

The retrieved articles were screened by two reviewers independently, firstly screening title and level and abstract, secondly reading the full text. The diagnostic criteria were developed from Rome criteria (including Rome I, II, III, and IV). Adult participants with IBS were included. Antispasmodics and eluxadoline were used as monotherapy or as adjunctive treatment in addition to usual care. All interventions lasted at least 1 week. No restrictions on the dosage of drugs and duration of treatment. RCTs for inflammatory bowel disease were excluded unless the IBS results were reported separately. Disagreement in the studies was resolved through group discussion and finally arbitrated by a third reviewer.

### 2.3 Outcome Assessments

The primary outcome was the relief of abdominal pain. The secondary outcome was the relief of global IBS symptoms (adequate relief of global IBS symptoms or FDA-recommended endpoints). Safety indicator was measured by evaluating the treatment-related adverse events. The included eligible RCTs often used different primary endpoints. However, some of the trials adhered to FDA-recommended endpoints and reported treatment efficacy according to a composite of improvement in both abdominal pain and stool consistency, or we were able to obtain these data from the original data.

### 2.4 Data Abstraction

Two reviewers (Q.F.T and S.L.H) extracted data independently on study characteristics through a standardized data extraction form. First, study characteristics were extracted including author’s name, article publication year, study design, sample size, participants’ age, gender, etc., Secondly, interventions were extracted including duration and dosage of the treatments, type of medicine. Thirdly, Outcome data were extracted—follow-up time points, mean and standard deviation (SD) for continuous data.

### 2.5 Risk of Bias Assessment

The risk of bias used the second version of the Cochrane risk of bias (RoB 2.0) to assessed (Sterne et al., 2019), which was updated in 2008. In RoB 2.0, the bias risk assessment was divided into five parts. Each part requires one or more questions to be answered, which leads to judgments of the risk of bias for a specific study (low, some concerns, or high risk of bias). Compared with the old version of the Cochrane risk-of-bias tool, the RoB 2.0 provides an overall rating of a study which facilitates judgment of the overall quality of the included trials.

### 2.6 Data Synthesis

We performed a network meta-analysis using a frequentist approach (Rücker, 2012). We calculated the treatment effect of one intervention as compared with the control and its standard error. All the outcomes were dichotomous. And then, we drew a net graph to summarized comparisons between two treatment categories and multiple individual-level treatments respectively. A p-score measures the mean probability of a treatment to be the most effective one, which is important in a network meta-analysis for the purpose of informing clinical practice. P-score can be easily calculated by one-sided *p*-values (Rücker and Schwarzer, 2015). The risk ratio (RR) and their corresponding 95% confidence intervals (95% CIs) between treatments in each outcome were estimated. A random-effects model was used for the network meta-analysis. The consistency of this study was examined through comparison among direct, indirect, and network estimates. We checked the significance of inconsistency by z test. The global heterogeneity of the network meta-analysis was calculated by global I^2^ statistics and tau-squared value. I^2^ >50% or a tau-squared value > 0.36 was considered as a sign of large heterogeneity. When a network meta-analysis has large heterogeneity, we further performed design-by-treatment analysis to detect the source of heterogeneity (König et al., 2013; Krahn et al., 2013).

## 3 Results

### 3.1 Trial Characteristics

**FIGURE 1:**
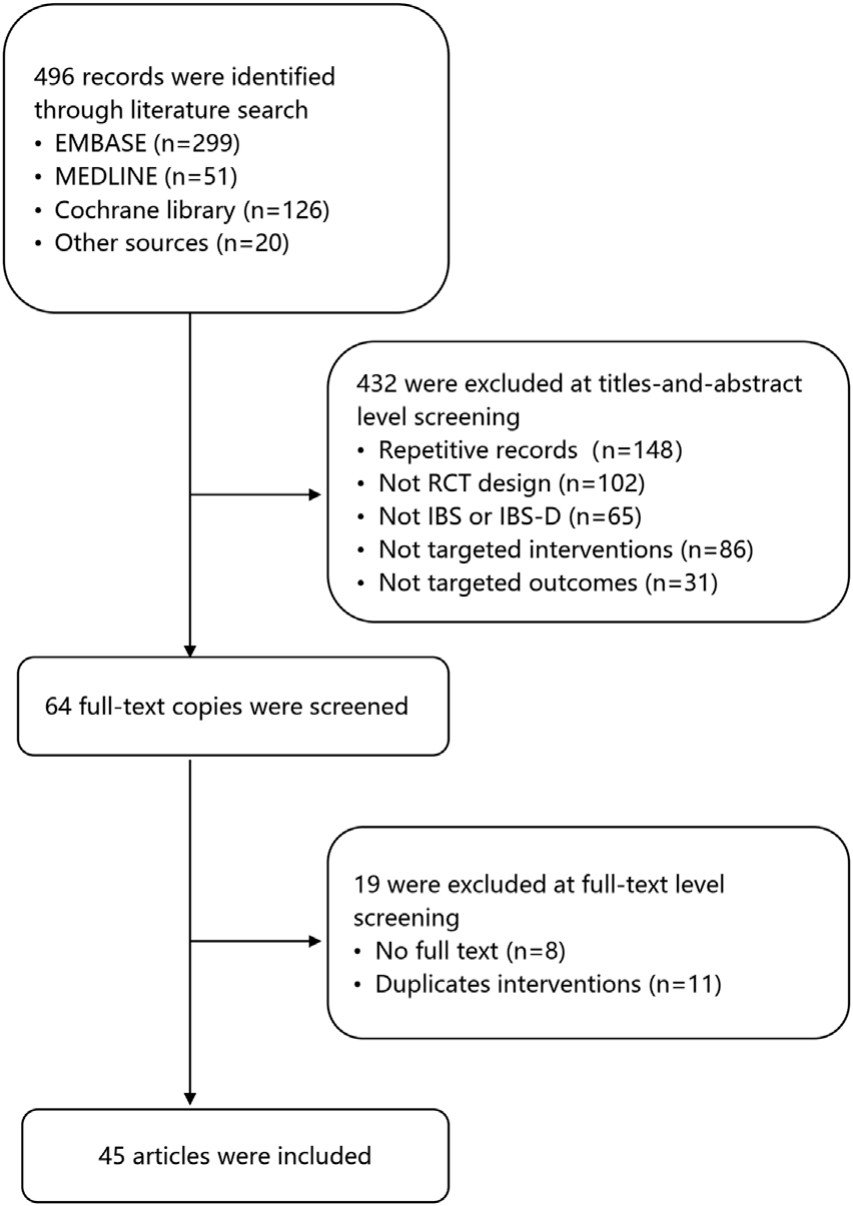
Study flow diagram.

The screening process of the review is shown in Figure 1. A total of 496 records were found after the first search. The second screening excluded 432 records, of which 148 were duplicates. 102 records were not RCT design, 65 records were not IBS, 117 records were not targeted interventions or outcomes. Nineteen studies were excluded at full-text level screening for unavailable full-text copies (*n* = 9) and duplicate outcomes (*n* = 11). A final total of 45 articles involving 42 RCTs and 8,457 patients were included for analysis (Figure 1). The median age of the subjects was 42 years old, and 62% of participants were female. Table 1 shows the characteristics of the patients in the included RCTs.

**TABLE 1:** Trial characteristics.

Author, year	Design	Sample	Age	Female (%)	Criteria	IBS subtype	Intervention	Time (wks)	Outcomes
Awad, 1995	NA	40	27.7	78	NA	NA	Pinaverium 50 mg od	3	Abdominal pain; symptom score
Baldi, 1992	Multicenter	71	40	60.60	NA	NA	Otilonium 40 mg tds	4	Abdominal pain
Battaglia, 1998	Multicenter	325	47.7	69	Rome I	NA	Otilonium 40 mg tds	15	Abdominal pain; global assessment
Brenner, 2019	Multicenter	346	43.9	70.2	Rome III	IBS-D	Eluxadoline 100 mg bid	12	Abdominal pain; adequate relief of global IBS symptoms
Chakraborty 2019	Single-center	40	35.6	75.0	Rome IV	IBS-D	Mebeverine 200 mg bid	8	Stool frequency; abdominal pain; IBS-QOL
Centonze, 1988	Single-center	48	NA	50	NA	NA	Cimetropium 50 mg tds	24	Abdominal pain; global assessment
Chang FY,2011	Single-center	117	53	71	Rome II	NA	Otilonium 40 mg tds	8	Abdominal pain; discomfort frequency score
Clavé P, 2011	Multicenter	356	46.6	71	Rome II	NA	Otilonium 40 mg tid	15	Abdominal pain; IBS symptom scale
Connell A, 1965	NA	40	40	63	NA	All subtype	Mebeverine 400 mg	12	Adverse effect; global assessment
D’Arienzo, 1980	NA	28	NA	39	NA	NA	Otilonium 20 mg tds	4	Symptom score
Delmont, 1981	Single-center	60	57	67	NA	NA	Pinaverium tds	4	Abdominal pain; global assessment
Dobrilla, 1990	Single-center	70	45	67	NA	All subtype	Cimetropium 50 mg tid	12	Global symptoms
Dubarry, 1977	Single-center	20	NA	NA	NA	NA	Pinaverium 50 mg tds	1	Abdominal pain
Dove, 2013	Multicenter	807	44.8	69.8	Rome III	IBS-D	Eluxadoline 5–200 mg bid	12	Abdominal pain; adequate relief of global IBS symptoms
Ducrotte 2014	Multicenter	436	54.4	47.4	Rome III	IBS-D	ACS tid	24	IBS-SSS; IBS-QOL
Everitt, 2013	Multicenter	135	44	80	Rome III	NA	Mebeverine 135 mg tid	6	IBS symptom scale; IBS-QOL
Fielding, 1980	NA	60	26	75	NA	NA	Trimebutine 200 mg tds	24	Abdominal pain; global assessment
Ghidini, 1986	Single-center	60	NA	60	NA	NA	Rociverine/Trimebutine tid	60 days	Abdominal pain
Gilvarry, 1989	NA	24	32	79	NA	NA	Pirenzepine 100 mg	4	Abdominal pain; global assessment
Glende, 2002	Multicenter	317	44	69	Rome I	NA	Otilonium 40 mg tid	15	Abdominal pain
Kruis, 1986	Single-center	80	41	61	NA	All substyle	Mebeverine 100 mg	16	Abdominal pain; global assessment
Levy, 1977	Single-center	50	48	46	NA	NA	Pinaverium 50 mg tds	2	Global assessment
Lüttecke, 1981	Single-center	40	45.3	53	NA	NA	Trimebutine 200 mg tid	3 days	Global symptoms
Lembo, 2016	Multicenter	2,428	45.2	66.1	Rome III	IBS-D	Eluxadoline 75/100 mg	52 or 26	Adequate relief of global IBS symptoms
Mitchell, 2002	Multicenter	107	53	80	Rome I	NA	Alverine 360 mg	12	Abdominal pain; global assessment
Moshal, 1979	Single-center	20	27	35	NA	NA	Trimebutine 200 mg tds	4	Abdominal pain
Page, 1981	Multicenter	97	36.7	83	NA	NA	Dicyclomine 40 mg qid	2	Abdominal pain; global assessment
Passaretti, 1989	Single-center	40	39	60	NA	NA	Cimetropium 50 mg tds	4	Abdominal pain; global assessment
Piai, 1979	Single-center	18	NA	56	NA	NA	Prifinium 30 mg tds	3	Global assessment
Pulpeiro, 2000	Single-center	85	45.2	69	NA	NA	Propinox 4 dd	4	Abdominal pain; global assessment
Rai,2014	Multicenter	180	46.5	13	Rome II	NA	Drotaverine 80 mg tid	4	Abdominal pain; bristol stool form scale
Ritchie, 1979	Single-center	96	38	74	NA	NA	Buscopan 10 mg qid	4	Global symptoms
Schafer, 1990	Multicenter	360	NA	NA	NA	NA	Butylscopamine 30 mg	4	Abdominal pain; global assessment
Secco, 1983	Single-center	30	45	50	NA	All subtype	Mebeverine 400 mg	4	Abdominal pain
Tasman-Jones C,1973	NA	24	43	58	NA	All subtype	Mebeverine 400 mg	4	Abdominal pain; global assessment
Virat, 1987	Multicenter	78	44	67	NA	NA	Pinaverium 50 mg tds	1	Abdominal pain; global assessment
Wittmann,2010	Multicenter	412	46.2	71	Rome III	NA	ACS tid	4	Abdominal pain; IBS symptom scale
Xing XC,2017	Single-center	114	43	65	Rome II	NA	Drotaverine 80 mg tid	4	Abdominal pain; stool frequency; SF-36
Yuan YZ,2005	Multicenter	160	NA	NA	Rome II	NA	Trimebutine 200 mg tid	4	Global assessment
Zheng L, 2015	Multicenter	427	36.7	55	Rome III	NA	Pinaverium 50 mg tid	4	Abdominal pain, bristol stool form scale
Zhong YQ,2007	Single-center	129	33	40	Rome III	IBS-D	Trimebutine 200 mg tid	4	Abdominal pain
Zhong YQ,2009	Single-center	82	36,6	52	Rome III	IBS-D	Alverine 60 mg bid	8	Abdominal pain

Notes: NA, not available; ACS, alverine citrate 60 mg + simeticone 300 mg.

The RoB2 showed that 10 (23.8%) RCTs were at low risk of bias in the overall assessment, whereas 31 (73.8%) trials presented some concerns. Only 1 (2.4%) trial had a high risk of bias. The overall assessment of RoB 2.0 is shown in Supplementary Figure S2.

### 3.2 Abdominal Pain

**FIGURE 2:**
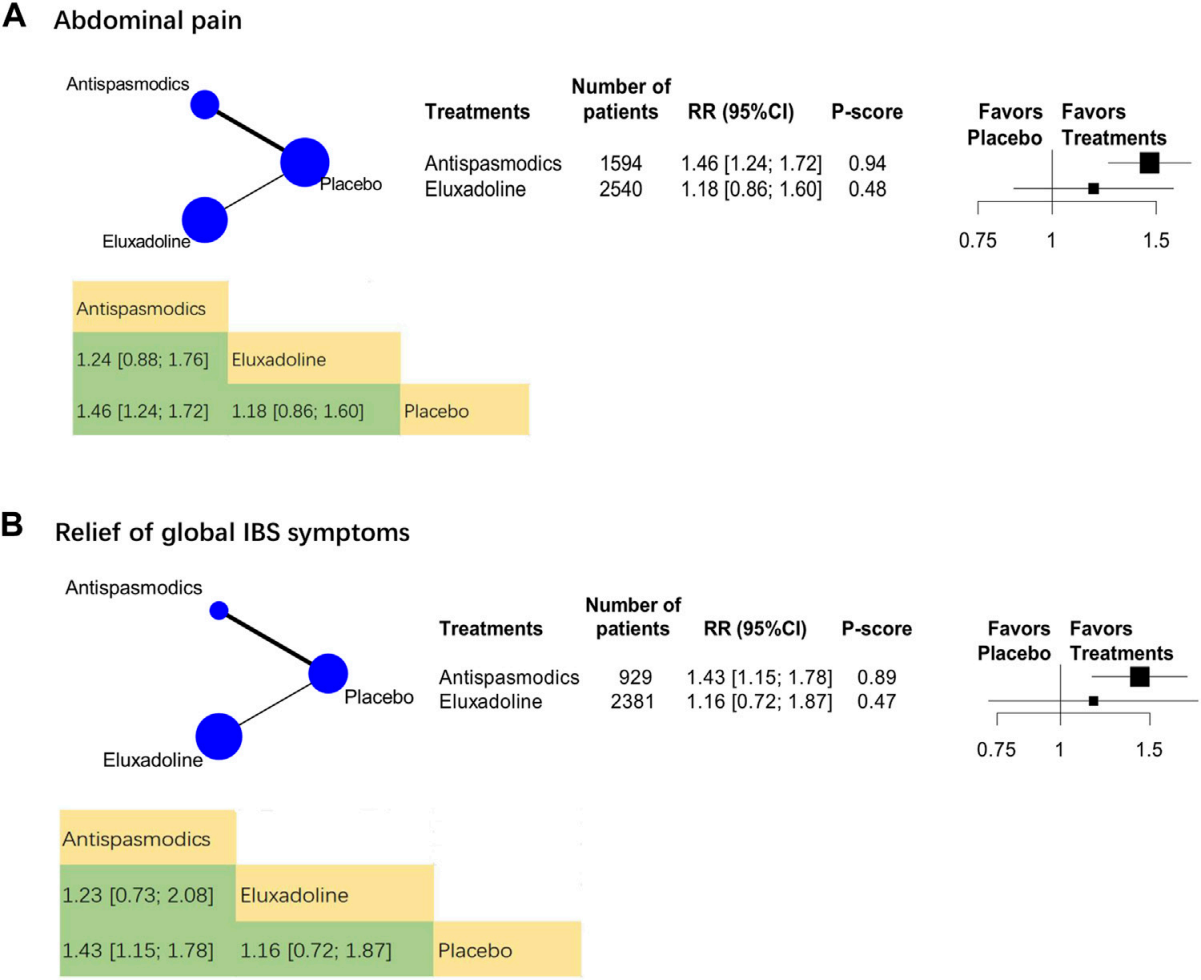
Category-level comparison of adequate relief of the relief of global IBS symptoms and abdominal pain. Subscript: Category-level analysis results for abdominal pain **(A)** and the relief of global IBS symptoms **(B)** were shown in this figure. The geometry of the networks is shown on the left. The size of the blue nodes corresponds to the number of participants assigned to treatments. The right shows the forest plots using placebo as a reference. Direct comparison links two treatments by a line; the thickness of the line corresponds to the number of trials that studied the treatment. P-scores are used to rank the effectiveness of each treatment. Treatments with the highest *p* values are the most effective. RR > 1 means this treatment superiority over placebo. Abbreviation: RR, risk ratio.

The analysis on efficacy against abdominal pain included 19 RCTs (*n* = 6,852). The category-level analysis assessed two treatment categories, whereas the individual-level analysis assessed 15 treatments. The categories analysis showed that, compared with placebo, antispasmodics were more effective [RR, 1.46 (95% CI, 1.24 to 1.72); P-score = 0.94; global I^2^ = 73.1%] (Figure 2A) but eluxadoline [RR, 1.18 (95% CI, 0.86 to 1.60)] was not.

**FIGURE 3:**
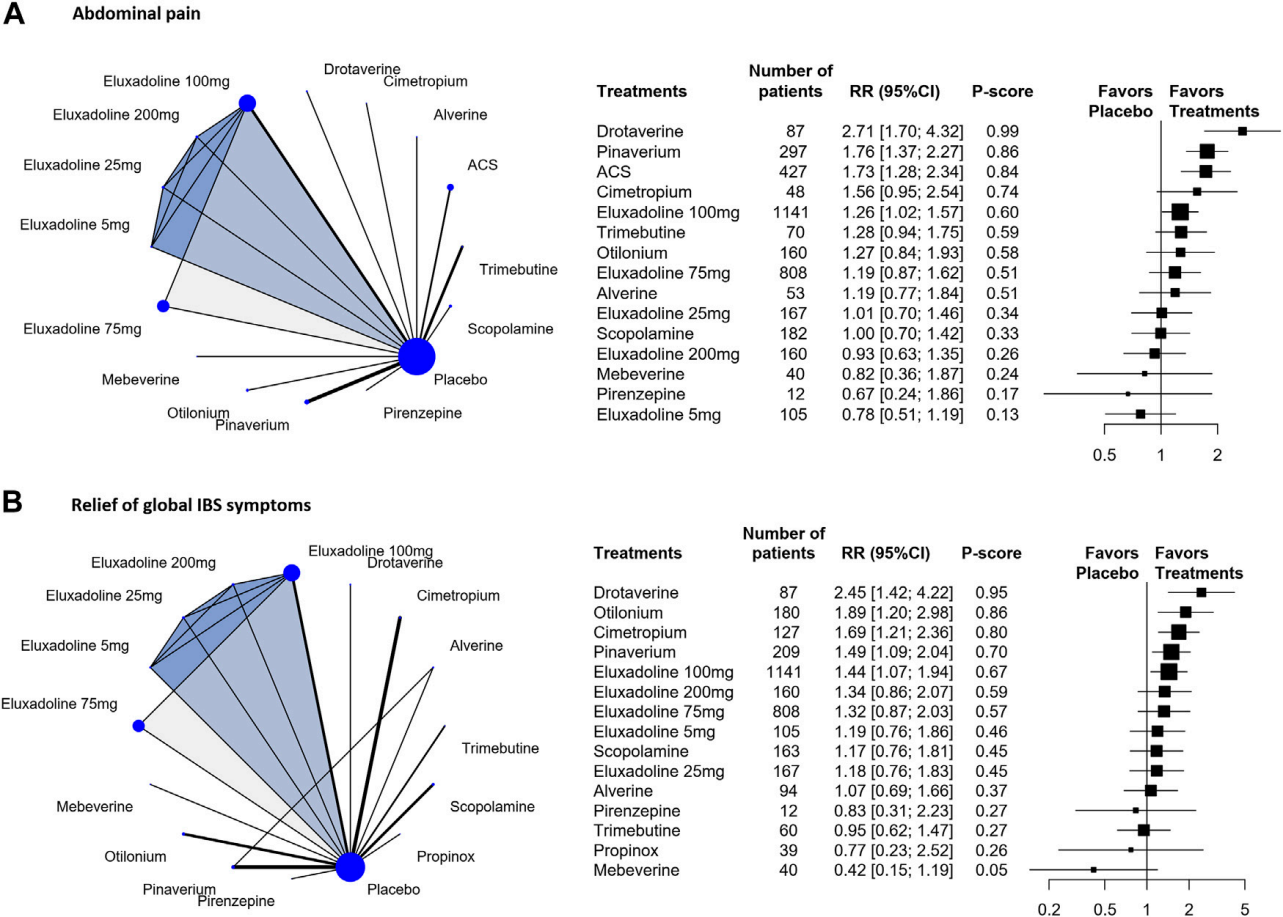
Individual-level comparison of adequate relief of the relief of global IBS symptoms and abdominal pain. Subscript: Individual-level analysis results for abdominal pain **(A)** and the relief of global IBS symptoms **(B)** were shown in this figure. The geometry of the networks is shown on the left. The size of the blue nodes corresponds to the number of participants assigned to treatments. The right shows the forest plots using placebo as a reference. Direct comparison links two treatments by a line; the thickness of the line corresponds to the number of trials that studied the treatment The blue or grey triangle among treatments indicates a three-arm design of an RCT. P-scores are used to rank the effectiveness of each treatment. Treatments with the highest *p* values are the most effective. RR > 1 means this treatment superiority over placebo. Abbreviation: RR, risk ratio.

The results of the individual-level analysis revealed drotaverine as the most effective [RR, 2.71 (95% CI, 1.70 to 4.32); P-score = 0.99; global I^2^ = 46.1%] (Figure 3A). Drotaverine, pinaverium, ACS, and eluxadoline 100 mg were more efficient than placebo (Figure 3A). Scopolamine, otilonium, pinaverium, ACS, and different doses of eluxadoline (75 and 100 mg) had similar effects on pairwise comparisons (Table 2). However, compared with ACS and pinaverium, eluxadoline 200 mg [RR, 0.53 (95% CI, 0.33 to 0.86)] had a significantly lower success rate in relieving abdominal pain. Furthermore, eluxadoline 100 mg [RR, 1.27 (95% CI, 0.84 to 1.93)] and 75 mg [RR, 1.19 (95% CI, 0.74 to 1.91)] showed slightly higher success rates than scopolamine (Table 2).

**TABLE 2:** Treatment estimate (comparison: different doses of eluxadoline VS antispasmodics).

Abdominal pain	—	—	N (n_1_/n_2_)	RR	95CI
Eluxadoline 100 mg	VS	Pinaverium	1,141/297	0.72	(0.51; 1.00)
Eluxadoline 200 mg	VS	Pinaverium	160/297	0.53	(0.33; 0.8)
Eluxadoline 75 mg	VS	Pinaverium	808/297	0.67	(0.45; 1.01)
Eluxadoline 100 mg	VS	ACS	1,141/427	0.73	(0.50; 1.06)
Eluxadoline 200 mg	VS	ACS	160/427	0.53	(0.33; 0.87)
Eluxadoline 75 mg	VS	ACS	808/427	0.68	(0.44; 1.06)
Eluxadoline 100 mg	VS	Otilonium	1,141/160	0.99	(0.62; 1.59)
Eluxadoline 200 mg	VS	Otilonium	160/160	0.73	(0.42; 1.28)
Eluxadoline 75 mg	VS	Otilonium	808/160	0.93	(0.55; 1.57)
Eluxadoline 100 mg	VS	Scopolamine	1,141/182	1.27	(0.84; 1.93)
Eluxadoline 200 mg	VS	Scopolamine	160/182	0.93	(0.55; 1.56)
Eluxadoline 75 mg	VS	Scopolamine	808/182	1.19	(0.74; 1.91)
**The relief of global IBS symptoms**					
Eluxadoline 100 mg	VS	Pinaverium	1,141/209	0.96	(0.64; 1.48)
Eluxadoline 200 mg	VS	Pinaverium	160/209	0.90	(0.52; 1.53)
Eluxadoline 75 mg	VS	Pinaverium	808/209	0.89	(0.52; 1.50)
Eluxadoline 100 mg	VS	Cimetropium	1,141/127	0.85	(0.54; 1.33)
Eluxadoline 200 mg	VS	Cimetropium	160/127	0.79	(0.46; 1.37)
Eluxadoline 75 mg	VS	Cimetropium	808/127	0.78	(0.46; 1.35)
Eluxadoline 100 mg	VS	Scopolamine	1,141/163	1.23	(0.72; 2.07)
Eluxadoline 200 mg	VS	Scopolamine	160/163	1.14	(0.62; 2.11)
Eluxadoline 75 mg	VS	Scopolamine	808/163	1.13	(0.62; 2.07)
**Adverse events**					
Eluxadoline 100 mg	VS	Pinaverium	1,142/398	1.13	(0.98; 1.31)
Eluxadoline 200 mg	VS	Pinaverium	172/398	1.19	(0.96; 1.48)
Eluxadoline 100 mg	VS	ACS	1,142/222	1.11	(0.85; 1.46)
Eluxadoline 200 mg	VS	ACS	172/222	1.17	(0.85; 1.60)
Eluxadoline 75 mg	VS	ACS	808/222	1.25	(0.84; 1.86)
Eluxadoline 100 mg	VS	Alverine	1,142/207	1.09	(0.94; 1.27)
Eluxadoline 200 mg	VS	Alverine	172/207	1.15	(0.92; 1.43)
Eluxadoline 75 mg	VS	Alverine	808/207	1.23	(0.88; 1.70)
Eluxadoline 100 mg	VS	Otilonium	1,142/555	1.13	(0.98; 1.29)
Eluxadoline 200 mg	VS	Otilonium	172/555	1.19	(0.96; 1.46)
Eluxadoline 75 mg	VS	Otilonium	808/555	1.27	(0.92; 1.75)
Eluxadoline 100 mg	VS	Hyoscine	1,141/182	1.22	(1.00; 1.47)
Eluxadoline 200 mg	VS	Hyoscine	172/182	1.28	(0.99; 1.64)
Eluxadoline 75 mg	VS	Hyoscine	808/182	1.07	(0.96; 1.94)

### 3.3 The Relief of Global IBS Symptoms

We analyzed 24 RCTs (*n* = 5,399) on their the relief of global IBS symptomss. The category-level analysis assessed two treatment categories, whereas the individual-level analysis assessed 15 treatments. The category-level results showed that antispasmodics were the most effective [RR, 1.43 (95% CI, 1.15 to 1.78); P-score = 0.89; global I^2^ = 86.7%] (Figure 2B). Furthermore, eluxadoline was less effective than antispasmodics (Figure 2B).

The individual-level results showed drotaverine was more effective than placebo [RR, 2.45 (95% CI, 1.42 to 4.22); P-score = 0.95] (Figure 3B). Drotaverine, otilonium, pinaverium, cimetropium, and eluxadoline 100 mg all showed superior the relief of global IBS symptomss over placebo (Figure 3B). Scopolamine, pinaverium, cimetropium, and different doses of eluxadoline (75, 100, and 200 mg) had similar effects on pairwise comparisons. In these comparisons, eluxadoline showed a slightly lower the relief of global IBS symptoms than pinaverium and cimetropium but a slightly higher rate than scopolamine (Table 2).

### 3.4 Sensitivity Analysis

Because RCTs on eluxadoline generally used the relief of global IBS symptoms as defined by the U.S. Food and Drug Administration, which required simultaneous improvement in the daily scores for the worst abdominal pain and stool consistency on the same day for at least 50% of the days assessed. The sensitivity analysis included 4 RCTs (*n* = 3,950). The category-level results showed that pinaverium [RR, 1.72 (95% CI, 1.33 to 2.21)] (Supplementary Table S1) was more effective than eluxadoline, whereas the individual-level results showed that pinaverium had the highest sensitivity rate [RR, 2.10 (95% CI, 1.67 to 2.65)]. Pinaverium and eluxadoline 100 and 75 mg were all superior over placebo (Supplementary Table S1).

### 3.5 Adverse Events

**FIGURE 4:**
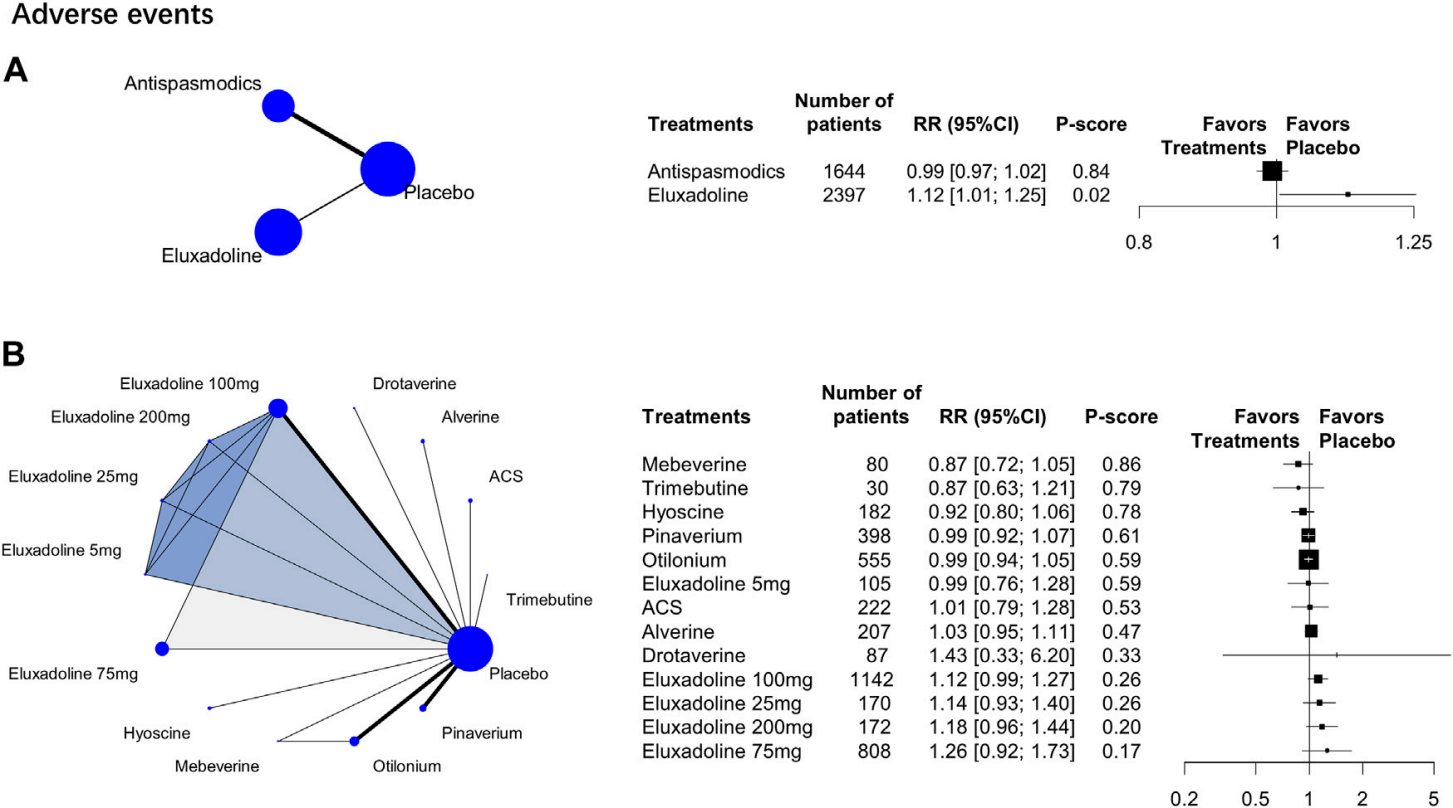
Treatment-related adverse events. Subscript: The figure shows category-level **(A)** and individual-level **(B)** analysis results of treatment-related adverse events. The geometry of the networks is shown on the left. The size of the blue nodes corresponds to the number of participants assigned to treatments. The right shows the forest plots using placebo as a reference. Direct comparison links two treatments by a line; the thickness of the line corresponds to the number of trials that studied the treatment The blue or grey triangle among treatments indicates a three-arm design of an RCT. P-scores are used to rank the effectiveness of each treatment. Treatments with the highest *p* values are the most effective. RR > 1 means this treatment superiority over placebo. Abbreviation: RR, risk ratio.

15 RCTs (*n* = 6,397) were analyzed. The category-level analysis assessed two treatment categories, whereas the individual-level analysis assessed 13 treatments. The category-level results showed that antispasmodics had lower adverse event rate [RR, 0.99 (95% CI, 0.97 to 1.02); P-score = 0.84; global I^2^ = 34.6%] than eluxadoline [RR, 1.12 (95% CI, 1.01 to 1.25)] (Figure 4A). But they were all higher than placebo.

The individual-level results showed that mebeverine ranked the best [RR, 0.87 (95% CI, 0.72 to 1.05); P-score = 0.86; global I^2^ = 34.6%] (Figure 4B). The adverse event rates of pinaverium, ACS, alverine, otilonium, and hyoscine all did not differ significantly from those of the different doses of eluxadoline (Table 2). However, eluxadoline 100 mg [RR, 1.22 (95% CI, 1.01 to 1.47)] had a significantly higher adverse event rate than hyoscine (Table 2). Of the different doses of eluxadoline, 100 mg had the lowest adverse event rate [RR, 1.12 (95% CI, 0.99 to 1.27); P-score = 0.26; global I^2^ = 34.6%] (Figure 3B), whereas 75 mg had the highest [RR, 1.26 (95% CI, 0.92 to 1.73); P-score = 0.17; global I^2^ = 34.6%] (Figure 3B).

In the study of antispasmodics, the most common adverse events were gastrointestinal symptoms like nausea, constipation, diarrhea, or bloating. These were mild, and no medical care was needed. However, in the study of eluxadoline, in addition to the common gastrointestinal symptoms, adverse events also included Pancreatitis, Spasm of the sphincter of Oddi (Lembo et al., 2016), and headache (Brenner et al., 2019).

## 4 Discussion

### 4.1 Main Findings

Our network meta-analysis showed that 1) eluxadoline 100 mg and some antispasmodics (drotaverine, pinaverium, ACS, otilonium, cimetropium) were effective in relieving abdominal pain and global IBS symptoms. 2) eluxadoline had more adverse event rates than antispasmodics. The primary outcome was the relief of abdominal pain. Because abdominal pain is a common symptom for most patients with IBS—regardless of type. And antispasmodics focused on the relief of abdominal pain. The secondary outcome was the relief of global IBS symptoms, including the severity and frequency of abdominal pain, the severity of abdominal distention, dissatisfaction with bowel habits, and interference with quality of life.

Our study used the adjusted indirect treatment comparison method to answer the clinical question: Is eluxadoline as effective as antispasmodics in relieving abdominal pain? A previous study has shown that the adjusted indirect treatment comparison method minimized the bias caused by variance in the treatment effect size and a multiarm design (Rücker, 2012). We found that the effect of eluxadoline on relieving IBS symptoms was not better than that of antispasmodics. This difference is clinically significant. To the best of our knowledge, the present study is the first network meta-analysis to compare eluxadoline with antispasmodics.

### 4.2 Comparison With Other Studies

Pharmacological treatments are the primary treatment option because they are convenient and effective. Systematic reviews and guidelines recommend antispasmodics as first-line pharmacologic treatments and have been used in the treatment of IBS for decades (Annaházi et al., 2014). A systematic review published in 2011 (Ruepert et al., 2011), concluded on the efficacy of antispasmodics as a treatment for IBS symptoms, especially abdominal pain. The individual subgroups included cimetropium/dicyclomine, pinaverium, and trimebutine.

Probiotics are also a treatment option in the treatment of IBS. Probiotics are attenuated bacteria, or bacterial products, that are beneficial to the host (Ford et al., 2017). Probiotics include food ingredients, such as fructose-oligosaccharides or inulin that promote the growth or activity of gut bacteria (Ford et al., 2014). There have been many RCTs of probiotics in IBS, the most effective probiotics included Bifidobacterium species and *Lactobacillus plantarum*. However, because of the various probiotics studied, there are some conflicting results among different trials. That limited the recommendations on which species or strain of probiotics is effective (Ford et al., 2018a). Therefore, probiotics were not included in our study.

Eluxadoline is a novel and expensive drug that was approved for the treatment of IBS-D in adults in Western countries in 2016. In 2019, eluxadoline was recommended for use as second-line therapy for improving IBS symptoms (Ford et al., 2020) at a prescribed dose of 75 mg or 100 mg twice daily (Cangemi and Lacy, 2019). In 2020, Black et al. (Black et al., 2020b) conducted a network meta-analysis to explore the efficacy of licensed pharmacological therapies for IBS-D or IBS-M often used as second-line therapy. Based on the U.S. Food and Drug Administration-recommended endpoint, they found eluxadoline 100 mg twice daily was significantly more effective than placebo in relieving global symptoms of IBS, abdominal pain, and diarrhea, whereas eluxadoline 75 mg twice daily was significantly more effective than placebo for global symptoms, but not more effective than placebo for abdominal pain. The results of the Black et al. study on eluxadoline 100 mg are consistent with our results, but not with that for eluxadoline 75 mg. We found eluxadoline 75 mg was no more effective than placebo in the two abovementioned outcomes.

### 4.3 Implications for Clinical Practice

Although eluxadoline has been proven effective for IBS in some studies (Dove et al., 2013b; Lembo et al., 2016; Cash et al., 2017; Keating, 2017; Özdener and Rivkin, 2017), several questions should be addressed before it could be widely used. First, our results show that commonly used first-line antispasmodics such as pinaverium and drotaverine are significantly better than eluxadoline in relieving abdominal pain in IBS. Moreover, eluxadoline is expensive and thus incurs a high medical cost. Future treatments of IBS may consist of antispasmodics in combination with other specific drugs. For example, pinaverium bromide combined with flupentixol-melitracen can effectively treat diarrhea-type IBS (Qin et al., 2019). Second, no evidence has been obtained on the long-term effects of eluxadoline after 2–3 months of and with continued treatment. This will need to be evaluated in future longitudinal studies. Third, the mechanism of action of eluxadoline is vaguely elucidated and presumed to be related to its unique combined κ- and δ-opioid receptor antagonist characteristics (Brenner et al., 2019). Further investigations into the pharmacological mechanism of eluxadoline are thus warranted. Fourth, our study showed eluxadoline had higher rates of adverse events compared with antispasmodics. Serious adverse events such as pancreatitis and sphincter of Oddi spasms were reported in previous trials (Dove et al., 2013a). Therefore, multiple safety concerns need to be satisfactorily addressed before using eluxadoline. Furthermore, eluxadoline is contraindicated for patients with biliary duct obstruction, severe liver problems, cholecystectomy, alcoholism, pancreatitis, sphincter of Oddi problems, and chronic or severe constipation. From 2015, when eluxadoline was initially approved, until 2017, 120 cases of pancreatitis were reported, some occurring after the initial dose. Of these, 76 resulted in hospitalization, two of which resulted in death (Pimentel, 2018). Thus, more long-term follow-up studies are needed to evaluate the safety of eluxadoline.

### 4.4 Study Limitations

This study has several limitations. First, the duration of treatment varied among the RCTs analyzed, and most studies lacked follow-up to assess long-term outcomes. Second, the network meta-analyses of the two primary outcomes and one safety outcome showed slightly greater heterogeneity but consistent results between direct and indirect estimates. The variety of antispasmodics, including dosages and usages, might be a source of heterogeneity and prevented a more comprehensive classification and analysis. Third, in some studies, eluxadoline would be used only after all other treatments (such as loperamide) have been undertaken. This indicates that patients in the eluxadoline groups in the trials had more severe conditions at baseline. In the final results, although the composite response rate of eluxadoline was higher than that of a placebo, no significant difference was found between the interventions. Therefore, some deviations may have occurred in the indirect comparison of antispasmodic agents. Fourth, some trials we included were old, the indicators and outcomes of its evaluation may introduce bias. Finally, eluxadoline has more serious adverse events, the most commonly reported are gastrointestinal symptoms including constipation, vomiting, and nausea. Severe patients can appear pancreatitis (Dove et al., 2013a; Cash et al., 2017).

## 5 Conclusion

Our study showed that some antispasmodics (e.g., drotaverine, pinaverium) and eluxadoline 100 mg are superiot to placebo in terms of improvement of abdominal pain and relief global IBS symptoms. But eluxadoline 100 mg has no advantage compare with those antispasmodics. Antispasmodics are still the first choice for the treatment of IBS. The safest dose of eluxadoline is 100 mg. However, eluxadoline had more reported adverse events and still requires long-term assessment in terms of safety and efficacy.

## Data Availability

The original contributions presented in the study are included in the article/Supplementary Material, further inquiries can be directed to the corresponding authors.
