# Supplementary material for: Eluxadoline Versus Antispasmodics in the Treatment of Irritable Bowel Syndrome: An Adjusted Indirect Treatment Comparison Meta-analysis
Source: Front Pharmacol. 2022 Feb 23;13:757969. doi: 10.3389/fphar.2022.757969 (PMC8906885; doi:10.3389/fphar.2022.757969)
Supplement: Supplementary file 1 [file DataSheet1.PDF]

eFigure 1. Overall risk of bias

Unique ID	Randomization process	Deviations from intended intervent	Missing outcome data	Measurement of the outcome	Selection of the reported result	Overall
Wittmann,2010	+	+	+	+	+	+
Mitchell,2002	+	+	?	+	+	!
Glende,2002	?	+	?	+	+	!
Clave,2011	+	+	?	+	+	!
Dove,2013	+	+	?	+	+	?
Brenner,2019	+	+	?	+	+	!
Ducrotte,2014	+	+	+	+	+	+
Chakraborty,2019	+	+	+	+	+	+
Battaglia,1998	+	+	?	+	+	!
Lembo,2016	+	+	+	+	+	+
Lembo,2018	+	+	+	+	+	+
Chey,2017	+	+	+	+	+	+
Page,1981	?	+	+	+	+	!
Chang,2011	?	+	+	+	+	!
Everitt,2013	+	+	+	+	+	+
Ramesh,2014	+	+	+	+	+	+
Xue,2017	+	+	+	+	+	+
Zheng,2015	+	+	+	+	+	+
Piai,1979	+	+	?	+	+	!
Ritchie,1979	?	+	+	+	+	!
Kruis,1986	?	+	?	+	+	!
Dobrilla,1990	?	+	+	+	+	!
Awad,1997	?	+	+	+	+	!
Gilvarry,1989	?	+	?	+	+	!
Centonze,1988	?	?	+	+	+	!
Schafer,1990	?	?	?	+	+	!
Passaretti,1989	?	+	?	+	+	!
Baldi,1992	?	?	+	+	+	!
Zhong,2009	?	?	+	+	+	!
Zhong,2007	?	?	?	+	+	!
Fielding,1980	?	+	+	+	+	!
Ghidini,1986	?	+	+	+	+	!
Yuan,2005	?	+	+	+	?	!

+ Low risk
? Some concerns
? High risk

eTable 1. Sensitivity analysis

Category-level analysis

Eluxadoline	1.72 [1.33; 2.21]	0.82 [0.74; 0.90]
0.58 [0.45; 0.75]	Pinaverium	0.48 [0.38; 0.60]
1.22 [1.11; 1.35]	2.10 [1.67; 2.65]	Placebo

Individual-level analysis

Eluxadoline 100mg	0.74 [0.58; 0.94]	0.81 [0.64; 1.01]	0.62 [0.45; 0.85]	0.95 [0.84; 1.08]	1.70 [1.31; 2.20]	0.81 [0.72; 0.90]
1.35 [1.06; 1.72]	Eluxadoline 200mg	1.09 [0.83; 1.44]	0.84 [0.59; 1.20]	1.29 [0.99; 1.68]	2.30 [1.64; 3.23]	1.09 [0.85; 1.40]
1.24 [0.99; 1.56]	0.92 [0.69; 1.21]	Eluxadoline 25mg	0.77 [0.55; 1.09]	1.18 [0.92; 1.52]	2.11 [1.52; 2.93]	1.00 [0.79; 1.27]
1.61 [1.17; 2.21]	1.19 [0.83; 1.70]	1.30 [0.92; 1.83]	Eluxadoline 5mg	1.53 [1.09; 2.14]	2.74 [1.84; 4.07]	1.30 [0.94; 1.79]
1.05 [0.93; 1.20]	0.78 [0.60; 1.02]	0.85 [0.66; 1.09]	0.65 [0.47; 0.91]	Eluxadoline 75mg	1.79 [1.37; 2.34]	0.85 [0.74; 0.97]
0.59 [0.45; 0.76]	0.43 [0.31; 0.61]	0.47 [0.34; 0.66]	0.37 [0.25; 0.54]	0.56 [0.43; 0.73]	Pinaverium	0.48 [0.38; 0.60]
1.24 [1.11; 1.38]	0.91 [0.71; 1.17]	1.00 [0.79; 1.26]	0.77 [0.56; 1.06]	1.17 [1.03; 1.34]	2.10 [1.67; 2.65]	Placebo

Footnotes: Comparisons between treatments should be read from left to right, and the comparison estimate is in the cell between the column-defining treatment and the row-defining treatment.

eTable 2. IBS Search strategy

OVID MEDLINE

1. randomised controlled trial.pt.
2. randomized controlled trial.pt.
3. controlled clinical trial.pt.
4. randomized.ab.
5. randomised.ab.
6. randomly.ab.
7. or/1-6
8. limit 7 to humans
9. exp Irritable Bowel Syndrome/
10. irritable bowel syndrome. ti,ab.
11. IBS. ti,ab.
12. IBS-D. ti,ab.
13. D-IBS. ti,ab.
14. or/9-13
15. eluxadoline.ab.
16. exp parasympatholytics/
17. parasympatholytics.ab.
18. mebeverine.ab.
19. alverine.ab.
20. pinaverium.ab.
21. rociverine.ab.
22. cimetropium.ab.
23. trimebutine.ab.
24. drotaverine.ab.
25. exp scopolamine derivatives/
26. scopolamine derivatives.ab.
27. butylscopolamine.ab.
28. hyoscine.ab.
29. exp trimebutine/
30. trimebutine.ab.
31. exp muscarinic antagonists/
32. muscarinic antagonists.ab.
33. pirenzepine.ab.
34. dicyclomine.ab.
35. otilonium.ab.
36. exp butylscopolammonium bromide/
37. butylscopolammonium bromide.ab.
38. or/15-37
39. 7 AND 14 AND 38

EMBASE

1. ‘randomized Controlled Trial’/exp
2. ‘randomized Controlled Trials as Topic’/exp
3. ‘randomized controlled trial’:ab,ti
4. ‘controlled clinical trial’/exp
5. ‘controlled clinical trial’:ab,ti
6. (#1 OR #2 OR #3 OR #4 OR #5) AND [humans]/lim
7. ‘Irritable Bowel Syndrome’/exp
8. ‘irritable bowel syndrome’:ab,ti
9. ‘IBS’:ab,ti
10. ‘IBS$’:ab,ti
11. #7 OR #8 OR #9 OR #10
12. ‘eluxadoline’/exp
13. ‘eluxadoline’:ab,ti
14. ‘parasympatholytics’/exp
15. ‘parasympatholytics’:ab,ti
16. ‘mebeverine’:ab,ti
17. ‘alverine’:ab,ti
18. ‘pinaverium’:ab,ti
19. ‘rociverine’:ab,ti
20. ‘cimetropium’:ab,ti
21. ‘trimebutine’:ab,ti
22. ‘drotaverine’:ab,ti
23. ‘scopolamine derivatives’/exp
24. ‘scopolamine derivatives’:ab,ti
25. ‘butylscopolamine’:ab,ti
26. ‘hyoscine’:ab,ti
27. ‘muscarinic antagonists’/exp
28. ‘muscarinic antagonists’:ab,ti
29. ‘pirenzepine’:ab,ti
30. ‘dicyclomine’:ab,ti
31. ‘otilonium’:ab,ti
32. ‘butylscopolammonium bromide’/exp
33. ‘butylscopolammonium bromide’:ab,ti
34. #12 OR #13 OR #14 OR #15 OR #16 OR #17 OR #18 OR #19 OR #20 OR #21 OR #22 OR #23 OR #24 OR #25 OR #26 OR #27 OR #28 OR #29 OR #30 OR #31 OR #32 OR #33
35. #6 AND #11 AND #34

Cochrane

1. (randomised controlled trial):ti,ab,kw
2. (irritable bowel syndrome):ti,ab,kw
3. (IBS):ti,ab,kw
4. (IBS-D):ti,ab,kw
5. #2 or #3 or #4
6. (eluxadoline):ti,ab,kw
7. (parasympatholytics):ti,ab,kw
8. (mebeverine):ti,ab,kw
9. (alverine):ti,ab,kw
10. (pinaverium):ti,ab,kw
11. (rociverine):ti,ab,kw
12. (cimetropium):ti,ab,kw
13. (trimebutine):ti,ab,kw
14. (drotaverine):ti,ab,kw
15. (scopolamine derivatives):ti,ab,kw
16. (butylscopolamine):ti,ab,kw
17. (hyoscine):ti,ab,kw
18. (trimebutine):ti,ab,kw
19. (muscarinic antagonists):ti,ab,kw
20. (pirenzepine):ti,ab,kw
21. (dicyclomine):ti,ab,kw
22. (otilonium):ti,ab,kw
23. (butylscopolammonium bromide):ti,ab,kw
24. #6 or #7 or #8 or #9 or #10 or #11 or #12 or #13 or #14 or #15 or #16 or #17 or #18 or #19 or #20 or #21 or #22 or #23
25. #1 and #5 and #24
